# Combining *TNF-α* silencing with *Wnt3a* overexpression: a promising gene therapy for particle-induced periprosthetic osteolysis

**DOI:** 10.3389/fcell.2025.1511577

**Published:** 2025-03-06

**Authors:** Ping Chen, Long Wu, Shuai Zhang, Qunhua Jin, Kening Sun

**Affiliations:** ^1^ Medical Experiment Center, General Hospital of Ningxia Medical University, Yinchuan, China; ^2^ Ningxia Key Laboratory of Clinical and Pathogenic Microbiology, General Hospital of Ningxia Medical University, Yinchuan, China; ^3^ Orthopedics Ward 3, The General Hospital of Ningxia Medical University, Yinchuan, China

**Keywords:** periprosthetic osteolysis, wear particle, gene therapy, TNF-α, Wnt3a

## Abstract

Wear particle-induced periprosthetic osteolysis is a prevalent issue that frequently leads to the failure of joint replacements, necessitating the development of effective therapeutic strategies. In this study, we established a mouse model of prosthetic loosening and evaluated the therapeutic effects of targeting tumor necrosis factor-alpha (*TNF-α*) and wingless-type MMTV integration site family, member 3A (*Wnt3a*) on osteolysis. *TNF-α* knockdown reduced inflammation and osteoclast-related gene expression, while *Wnt3a* overexpression increased osteoblast-related gene expression. Notably, the combination of these interventions showed superior efficacy in inhibiting osteolysis compared to monotherapy. Biomechanical imaging and histological staining revealed that combined therapy enhanced bone density and minimized the gaps between the peri-prosthetic bone and the prosthesis, reducing fibrous connective tissue proliferation. Adeno-associated virus-mediated gene therapy was found to be safe, with no adverse effects observed in liver, brain, spleen, and kidney tissues. Our findings suggest that combining *TNF-α* silencing with *Wnt3a* overexpression may be a promising approach for treating particle-induced peri-implant osteolysis and warrants further clinical investigation.

## 1 Introduction

Total joint arthroplasty (TJA) is a cornerstone treatment for end-stage painful joint diseases, yet it is not without complications, particularly aseptic prosthesis loosening (APL), which is a leading cause of failure post-TJA (Bozic et al., 2010; Howie et al., 2021; Rozell et al., 2021). The pathogenesis of APL is multifactorial, with wear particles from prosthetic devices triggering macrophage-mediated inflammatory responses that disrupt bone homeostasis (Amirhosseini et al., 2018; Bohara and Suthakorn, 2022). Despite advancements in wear-resistant materials (Mao et al., 2024), surgical techniques (Cilla et al., 2017), and pharmacological interventions (Wu et al., 2021), APL remains a significant challenge, highlighting the need for more effective therapies.

Among the inflammatory cytokines, tumor necrosis factor-alpha (*TNF-α*) plays a critical role. *TNF-α*, through its interaction with transmembrane TNF receptor superfamily, member 1a (*TNFR*), controls cell survival or induces apoptosis via complex signal pathways (Pacheco et al., 2014). It is a key mediator in the pathogenesis of APL, modulating osteoblast-associated receptors to increase the expression of receptor activator of nuclear factor (NF)-κB ligand (RANKL), thus facilitating osteoclastogenesis (Komine et al., 2001). Moreover, *TNF-α* can directly induce macrophage differentiation into osteoclasts in a RANKL-independent manner, highlighting its significance in the osteolytic process (Guo et al., 2013; Sun et al., 2013).

Wnt signaling pathway also plays an important role in the pathophysiological process of osteolysis. Wingless-type MMTV integration site family, member 3A (*Wnt3a*), a member of the Wnt family of secreted glycoproteins, is crucial in bone formation (Byun et al., 2014). It acts as a key modulator of mesenchymal stem cell (MSC) differentiation, guiding the lineage commitment towards osteoblastogenesis, and facilitating bone matrix deposition and mineralization (Li et al., 2022). The canonical *Wnt3a* pathway involves its binding to a receptor complex comprising Frizzled (Fz) receptors and low-density lipoprotein receptor-related proteins 5/6 (LRP5/6), which activates intracellular Disheveled (Dvl) proteins, leading to the stabilization and nuclear translocation of β-catenin (Bourhis et al., 2010). *Wnt3a*′s role in bone formation is further underscored by its regulatory influence on the balance between bone resorption and formation, a critical process in maintaining skeletal homeostasis (Lu et al., 2008; Rawadi et al., 2003).

Earlier studies indicated that *TNF-α* can suppress the expression of *Wnt3a* by activating the NF-κB signaling pathway, thereby leading to impaired osteoblast function (Majumder et al., 2012; Takada et al., 2023). Additionally, the activation of *Wnt3a* can inhibit *TNF-α*-induced interleukin 6 (*IL-6*) synthesis, indicating the anti-inflammatory role of *Wnt3a* in regulating bone metabolism (Chaganti et al., 2014). In the context of bone resorption, modulating the levels of *TNF-α* or enhancing the expression of *Wnt3a* may help to slow down the process of bone resorption and improve bone health (Liao et al., 2020). Despite the individual roles of *Wnt3a* and *TNF-α* being well-demonstrated, there is a lack of research on their combined impact, particularly in prosthetic loosening.

This study introduces a novel application of gene therapy in the context of APL, where an adeno-associated virus (AAV) carrying *TNF-α*-siRNA and *Wnt3a* is locally administered in a mouse tibial prosthesis osteolysis model. Our approach aims to provide a comprehensive analysis of the effects on osteolysis and bone formation, potentially offering a new therapeutic strategy for APL.

## 2 Materials and methods

### 2.1 Human knee joint sample collection and processing

Human knee joint sample were collected from patients undergoing primary TJA and revision TJA due to aseptic loosening at the General Hospital of Ningxia Medical University. The collection and use of human sample were conducted in strict compliance with ethical guidelines and were approved by the Institutional Review Board of the General Hospital of Ningxia Medical University (Ethical Approval No. 2020-913).

Primary TJA sample were collected from patients undergoing primary TJA for end-stage osteoarthritis or other degenerative joint diseases, served as control. Revision TJA sample were collected from patients undergoing revision TJA due to aseptic loosening caused by wear particle-induced osteolysis. Immediately after collection, the clinical sample were rinsed with sterile phosphate-buffered saline (PBS) to remove blood and debris. The tissue was snap-frozen in liquid nitrogen and stored at −80°C for RNA isolation.

### 2.2 Titanium particle preparation

Titanium alloy particles (Ti–6Al–4V, the medium particle size 5 μm, range 4–6.35 µm) used in this project were generated by the Zimmer Corporation (Indiana, United States). Sterilization was achieved by heating the particles at 180°C for 45 min. They were then cleaned with three ethanol rinses to remove contaminants, followed by three phosphate-buffered saline (PBS) rinses to neutralize the surface. Endotoxin levels were verified to be below 0.1 EU/mL using the E-Toxate kit from Sigma. For *in vivo* applications, a suspension was made by dispersing 10 mg of particles in 1 mL of normal saline (Yu et al., 2013).

### 2.3 Construction of adeno-associated virus vectors (AAV)

siRNA oligos (5′-GGT​TGC​CTC​TGT​CTC​AGA​ATT-3′) targeting *TNF-α* were synthesized and annealed by Sangon Biotech (Shanghai, China). Then, siRNA oligos were cloned into the pAdEasy-U6-CMV-EGFP vector. The full-length *Wnt3a* cDNA from the pCDNA3.1 vector was cloned into the pAdEasy-EF1-MCS-3flag-CMV-EGFP vector using KpnI and XhoI restriction sites. Positive clones were confirmed by PCR and sequencing. Recombinant adenoviral vectors were created by recombination between pAdEasy-EF1-*Wnt3a*-3flag-CMV-EGFP and pAdeasy-1 in *E. coli* BJ5183. For dual expression of *Wnt3a* and *TNF-α*-siRNA, pHBHD-U6-*TNF-α*-siRNA-CMV-*Wnt3a* was linearized with PacI and co-transformed with pAdeasy-1 into *E. coli* BJ5183. Adeno-associated virus packaging and purification were conducted by Hanheng Biological (Shanghai, China).

### 2.4 Wear particle-induced knee prosthesis failure model

60 SPF-grade BALB/c mice (10-week-old, 28–30 g) were purchased from Chongqing Enswell Biotechnology (Chongqing, China) and maintained under standard conditions for an additional 2 weeks. The mice were divided into the following five groups, with 12 mice in each group: negative control group (PBS injection + titanium nails), positive control group (titanium particle suspension + titanium nails), *TNF-α*-siRNA treatment group (titanium particle suspension + titanium nails + *TNF-α*-siRNA), *Wnt3a* overexpression group (titanium particle suspension + titanium nails + *Wnt3a* overexpression) and combination therapy group (titanium particle suspension + titanium nails + *TNF-α*-siRNA-*Wnt3a* overexpression).

Prior to surgery, mice were fasted but provided water *ad libitum*. Anesthesia was induced with an intraperitoneal injection of 10% chloral hydrate at 30 mg/kg (Liu et al., 2019). The surgical site’s fur was removed, and the skin was disinfected with povidone-iodine solution. A 5 mm medial parapatellar incision was made to expose the patellar ligament and tibial plateau. A bone tunnel was drilled into the tibial plateau using a 0.8 mm dental burr, extending 5 mm into the medullary cavity from the center of the plateau.

Negative control group mice received a 10 µL PBS injection into the tunnel, while Positive control group and the gene therapy group was treated with a 10 µL suspension of titanium particles at 10 mg/mL. Artificial prosthetic titanium nails were then implanted in all mice, ensuring the nail cap was flush with the tibial plateau’s articular surface. Post-implantation, joint mobility was assessed for range of motion and stability, and the incision was closed in layers. Postoperative X-rays confirmed the prosthesis’s positioning within the bone marrow cavity.

All mice were screened for common pathogens, including mouse hepatitis virus, minute virus of mice, mouse parvovirus, and others, to ensure specific pathogen-free conditions. The animal study was approved by the Animal Research-Animal Care Committee of the General Hospital of Ningxia Medical University (Ethical Approval No. 2020-913, same as the human study).

### 2.5 Micro-computed tomography (µCT) analysis

Tibiae with implanted titanium nails underwent high-resolution micro-computed tomography (micro-CT) using the SuperNova®CT SNC-100 system (PINGSEGN). The scanning parameters were set at 15 μm per layer with a voltage of 45 kV and a current of 435 μA. A region of interest (ROI), 1.7 mm in diameter and adjacent to the tibial growth plate, was selected for analysis. Three-dimensional (3D) reconstruction of the images was performed to assess morphometric parameters, including bone mineral density (BMD) and bone volume fraction (BV/TV).

### 2.6 AAV-mediated gene therapy for the treatment of knee prosthesis osteolysis

Starting from the second week postoperatively, mice in the study received a series of biweekly intra-articular injections of 40 µL into the operated limbs. Both the negative and positive control groups were injected with PBS. The treatment group was administered *TNF-α*-siRNA, OE-*Wnt3a*, and *TNF-α*-siRNA-OE-*Wnt3a* adeno-associated virus at a concentration of 2 × 10^10 pfu/mL. This injection regimen was continued for 16 weeks post-surgery, with all mice maintained on a standard diet throughout the study period.

### 2.7 Enzyme-linked immunosorbent assay (ELISA)

Tissue samples were collected from areas surrounding the implants at 4, 8, 12, and 16 weeks post-implantation for *TNF-α*, *Wnt3a* and urine samples were obtained at 16 weeks for urinary deoxypyridinoline (DPD). *TNF-α* level was measured using ELISA kit from Ruixinbio (Quanzhou, China). *Wnt3a* and DPD level was measured using ELISA kit from COIBO BIO (Shanghai, China). All procedures were performed following the manufacturers' instructions.

### 2.8 Total RNA extraction and quantitative real-time PCR (q-PCR)

Total RNA was extracted from peri-prosthetic tissue, which included both bone tissue and associated soft tissues surrounding the prosthesis. The tissues were homogenized in TRIzol reagent (Invitrogen, United States) using a grinding machine (Cebo, CEBO-24) with steel beads. RNA was isolated following the protocol, which involved phase separation with chloroform, RNA precipitation with isopropanol, and washing with 75% ethanol. The RNA was dissolved in RNase-free water, and its concentration and purity were measured using a NanoDrop spectrophotometer (Thermo Fisher Scientific, United States), with A260/A280 ratios between 1.8 and 2.0 indicating high-quality RNA.

Subsequently, 1 μg of RNA was reverse transcribed into complementary DNA (cDNA) using the HiScript II Reverse Transcriptase Kit (Noweizan, China). q-PCR was then performed on the ABI 7900 real-time PCR system to analyze the cycle threshold values. The specific sequences of the PCR primers used in this study are detailed in Supplementary Table S1.

### 2.9 Western blot

Peri-prosthetic tissues, including both bone and associated soft tissues, were homogenized using a grinding machine (Cebo, CEBO-24) with steel beads in RIPA buffer supplemented with protease inhibitors. The homogenization involved grinding, followed by vortexing for 1 min and incubation on ice for 10 min. The homogenate was centrifuged at 13,000 rpm at 4°C for 20 min, and the supernatant was collected. Proteins (500 µg) were mixed with 5× SDS loading buffer in a 4:1 ratio and denatured at 100°C for 6 min. Samples (30 µg per lane) were resolved by 12% SDS-PAGE and transferred onto the PVDF membrane, which was blocked with 5% non-fat milk for 1 h at room temperature. The membrane was incubated overnight at 4°C with primary antibodies against RANKL (abclon, A2550), osteocalcin (OCN) (abclon, A20800), osteoprotegerin (OPG) (abclon, A2100), tartrate-resistant acid phosphatase (TRAP) (abclon, A0962), and glyceraldehyde-3-phosphate dehydrogenase (GAPDH) (abclon, A19056) at a 1:1000 dilution. After washing, the membrane was incubated with Goat anti-Rabbit IgG secondary antibody (Abclonal, AS014) for 1 h at room temperature. Protein bands were visualized using an ECL detection reagent (Yeasen, China) for 1 min and imaged using Fusion FX6 Spectra imaging system.

### 2.10 Histological staining

Periprosthetic tissues (including both bone and associated soft tissues) and major organs (liver, spleen, kidney, and brain) were collected and fixed in 4% paraformaldehyde. For bone tissues, decalcification was performed using 10% formic acid for 7–10 days, with the solution changed daily, until the bones were sufficiently softened. After decalcification, the bone tissues were rinsed thoroughly with distilled water to remove residual acid. Subsequent processing for both bone and soft tissues included dehydration through a graded ethanol series (70%, 80%, 90%, and 100%), clearing in xylene, and paraffin embedding. The samples were then sectioned, deparaffinized, and rehydrated before being stained with hematoxylin and eosin (H&E) using a commercial kit from Servicebio (Wuhan, China). Following staining, the slides were dehydrated, cleared, and mounted with neutral resin. Histological evaluation was conducted using an Mshot MF53 microscope.

### 2.11 Goldner staining

Tibial bone specimens were collected from mice at 16 weeks post-surgery and processed for Goldner staining using a kit from Servicebio (Wuhan, China) following the manufacturer’s instructions. The specimens were fixed in 4% paraformaldehyde for 48 h and then subjected to a series of histological procedures including dehydration, clearing, wax impregnation, and paraffin embedding to prepare paraffin sections. These sections were deparaffinized and rehydrated before staining.

The staining protocol involved incubating the slides with an equal volume mixture of Goldner A/B solutions for 20 min, followed by differentiation in 1% acid alcohol. Subsequently, the slides were immersed in Goldner C solution for 10 min, rinsed briefly in 0.2% acetic acid, treated with Goldner D solution for 3 min, and finally stained with Goldner E solution for 5 min. After staining, the sections were dehydrated through a graded ethanol series and xylene, mounted with neutral resin, and visualized using a 3DHISTECH Pannoramic MIDI slide scanner and corresponding software.

### 2.12 Von kossa staining

Tibial bone sections were prepared for Von Kossa staining, paralleling the process for Goldner staining. The Von Kossa staining solution from Servicebio (Wuhan, China) was applied, and the Rehydrated sections were covered and exposed to ultraviolet (UV) light for 15 min. The slides were then washed extensively with distilled water. Following this, the sections were stained with hematoxylin for 3 min, differentiated, and then rinsed with running water to develop the blue color. A series of dehydration steps in graded ethanol was performed, after which eosin staining was applied. The sections were then dehydrated through anhydrous ethanol and xylene before being mounted with neutral resin. The stained sections were visualized using a 3DHISTECH Pannoramic MIDI slide scanner.

### 2.13 Biomechanical testing

After the humane sacrifice of the animal, the mouse limb retaining the intact implant was extracted by disarticulating the knee joint. Subsequently, all surrounding soft tissues were meticulously dissected to reveal the surface of the implanted pin and the proximal tibia. The exposed tissue was securely mounted onto a custom fixture. Multiple nylon threads were then wound around the titanium nail cap, with their opposite ends encircling a screw, which was anchored to the fixture of a tensile testing machine. The setup was meticulously aligned to ensure that the pin’s longitudinal axis was parallel to the direction of extraction and perpendicular to the tension applied by the nylon threads. A controlled tension test was then executed at a displacement rate of 1.0 mm/min, continuing until the titanium nail was completely extracted. Throughout this process, the load in newtons (N) and displacement in millimeters (mm) were meticulously documented for subsequent analysis.

### 2.14 Statistical analysis

Statistical analysis and graph plotting were performed using SPSS 20.0 and Prism 8.0 software. Each experiment included three biological replicates, and data are presented as mean ± standard deviation. One-way ANOVA followed by Sidak’s *post hoc* test was used for multiple group comparisons with homogeneity of variances, and Dunnett’s T3 test was used when variances were not homogeneous. Statistical significance was accepted at p < 0.05 for all analyses.

## 3 Results

### 3.1 *TNF-α* and *Wnt3a* gene expression in periprosthetic tissue

**FIGURE 1:**
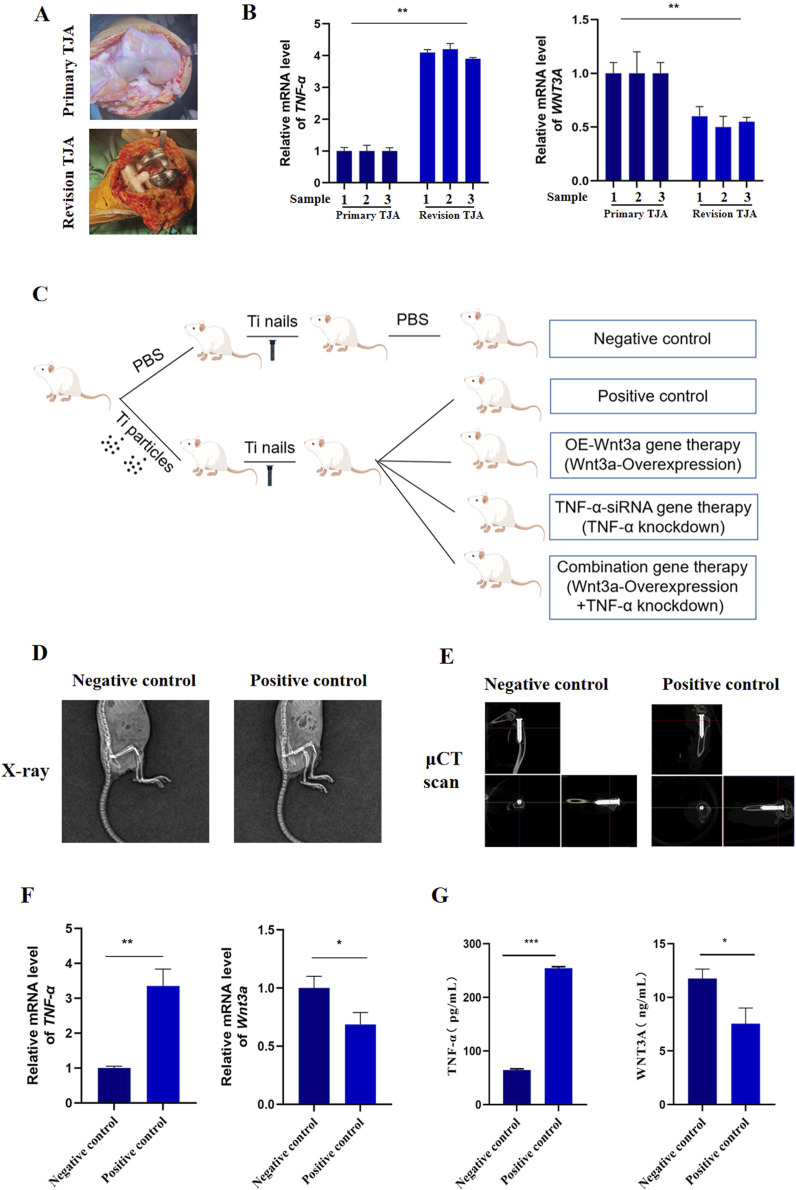
The expression of *Wnt3a* and TNF-α genes in periprosthetic tissue **(A)** Clinical samples of primary and revision tissues after total joint arthroplasties **(B)** mRNA levels of TNF-α and *WNT3A* in primary and revision tissues of total joint arthroplasties **(C)** Schematic diagram of the construction process of mouse knee prosthesis model **(D)** Immediate post-operative radiograph **(E)** Immediate postoperative μCT scan **(F)** mRNA levels of *TNF-α* and *Wnt3a* in particle-induced implant loosening model **(G)** Protein levels of TNF-α and *WNT3A* in particle-induced implant loosening model.

To elucidate the roles of *TNF-α* and *Wnt3a* in aseptic loosening of prostheses, we collected tissue samples from patients undergoing primary total joint arthroplasty (TJA) and those experiencing aseptic loosening requiring revision TJA (Figure 1A). q-PCR analysis revealed significantly elevated *TNF-α* expression in revision TJA tissues compared to primary TJA tissues. In contrast, *WNT3A* gene expression was lower in revision TJA tissues (Figure 1B; Supplementary Figure S1A). These findings suggest a potential involvement of *TNF-α* and *WNT3A* in wear-related processes associated with aseptic loosening.

We then developed a murine model of aseptic loosening of knee prostheses and conducted gene therapy to explore the roles of *TNF-α* and *Wnt3a* in this condition (Figure 1C; Supplementary Figure S1B). Postoperative X-ray and μCT examination confirmed the accurate implantation of the titanium nail into the medullary cavity (Figures 1D, E). q-PCR analysis showed that *TNF-α* expression in the positive control group was significantly higher than in the negative control group, while *Wnt3a* expression was markedly lower (Figure 1F). These results were corroborated by ELISA results (Figure 1G). Briefly, these results indicate that our model mouse reflects the expression of *TNF-α* and *Wnt3a* genes observed in clinical samples.

### 3.2 Knockdown of *TNF-α* suppressed osteoclastogenesis

**FIGURE 2:**
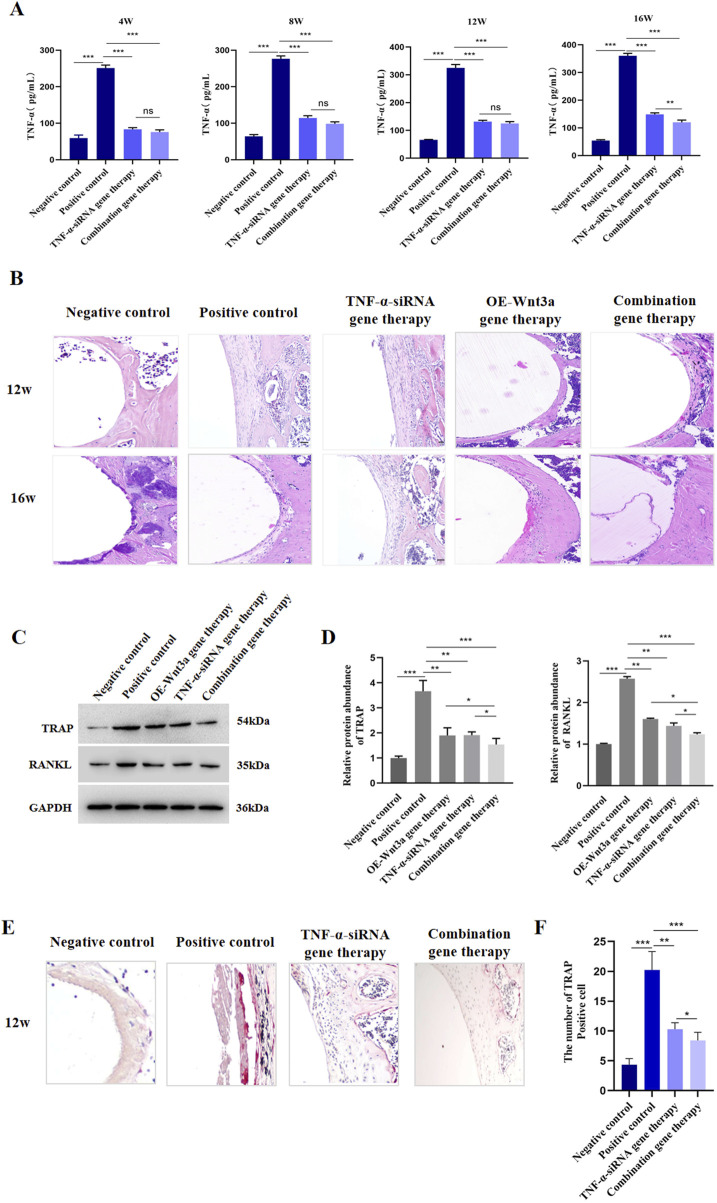
TNF-α knockdown suppressed osteoclastogenesis **(A)** ELISA detection of TNF-α protein level in tissues around prosthesis at different time points **(B)** H&E staining of the synovial tissue around the prosthesis **(C)** WB detection of protein expression levels of TRAP and RANKL after TNF-α gene therapy **(D)** Statistical results of gray value quantification **(E)** Immunohistochemical staining for TRAP **(F)** Quantitative analysis of TRAP staining.

To investigate the effect of *TNF-α* on osteoclast differentiation, we injected AAV carrying *TNF-α*-siRNA into the knee joint cavity and detected the expression of *TNF-α* in tissue surrounding the prosthesis. ELISA data revealed a significant suppression of *TNF-α* gene expression in the *TNF-α*-siRNA-treated group compared to the positive control group at different time points (Figure 2A). Additionally, to confirm the specificity of *TNF-α* knockdown and rule out potential off-target effects, we assessed the mRNA levels of the top predicted off-target genes in the RAW264.7 cell model stimulated with titanium particles. As shown in Supplementary Figure S1C, no significant changes in the expression levels of these off-target genes were observed following *TNF-α* knockdown, confirming the specificity of the siRNA-mediated *TNF-α* suppression. Histological examination through H&E staining of peri-prosthetic tissues showed that at 12 weeks, the negative control group displayed minimal inflammatory cell infiltration, no distinct boundary membranes, and well-preserved bone structures (Figure 2B). In contrast, the positive control group exhibited extensive inflammatory cell infiltration, substantial membrane thickening, and significant bone destruction. Notably, the *TNF-α*-siRNA treated group demonstrated minimal inflammatory cell infiltration, thin membrane formation, and no significant bone destruction (Figure 2B). Over time, the positive control group showed an escalation in inflammatory cell infiltration, while the *TNF-α*-siRNA treatment group showed a relative decrease in inflammatory cells and a reduction in boundary membrane thickness.

Given that RANKL and TRAP are pivotal in osteoclastogenesis, we next evaluated the effects of *TNF-α* knockdown on their expression. Western blot analysis indicated that *TNF-α* knockdown resulted in reduced levels of both RANKL and TRAP (Figures 2C,D; Supplementary Figure S2). Since *TNF-α* is known to regulate the production of other inflammatory cytokines, including *IL-6*, we also measured *IL-6* expression post-therapy. Consistent with our hypothesis, *IL-6* expression was downregulated following *TNF-α* knockdown (Supplementary Figure S3). TRAP staining, used to visualize differentiated osteoclasts, showed a significantly higher number of TRAP-positive cells in the positive control group, while the *TNF-α* knockdown and negative control groups displayed fewer TRAP-positive cells (Figures 2E, F). In line with these findings, genes associated with osteoclast formation and function, such as nuclear factor of activated T cells, cytoplasmic, calcineurin dependent 1 (*Nfatc1*), *Trap*, osteoclast associated receptor (*Oscar*), matrix metallopeptidase 9 (*Mmp9*), and cathepsin K (*Ctsk*), were also downregulated following *TNF-α* knockdown (Supplementary Figure S3). Collectively, these results suggest that AAV-mediated *TNF-α* knockdown effectively reduces osteoclast formation in prosthetic model mice.

### 3.3 Overexpression of *Wnt3a* promoted osteoblast differentiation

**FIGURE 3:**
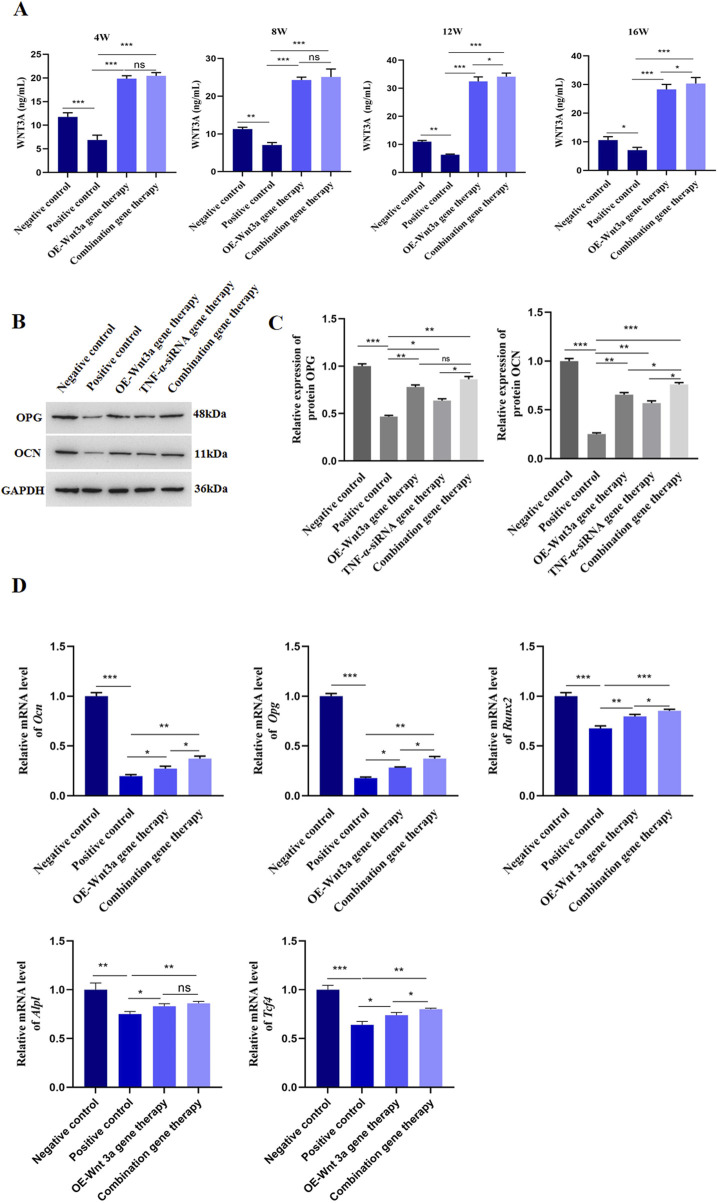
Overexpression of *Wnt3a* promoted osteoblast differentiation **(A)** ELISA detection of WNT3A protein level in tissues around prosthesis at different time points **(B)** WB detection of protein expression levels of OCN and OPG after *Wnt3a* gene therapy **(C)** Statistical results of gray value quantification **(D)** q-PCR detection of mRNA expression levels of *Ocn*, *Opg*, *Runx2*, *Alpl* and *Tcf-4* after *Wnt3a* gene therapy.

To elucidate the role of *Wnt3a* in osteoblast differentiation, we employed an AAV vector to overexpress *Wnt3a* in mouse models. We subsequently measured *Wnt3a* gene expression in peri-prosthetic tissues at various time points following gene therapy. ELISA results demonstrated a significant upregulation of *Wnt3a* in the treatment group compared to the positive control, validating the efficacy of our AAV-mediated approach in enhancing *Wnt3a* expression levels (Figure 3A). Subsequent H&E staining around the prosthesis revealed minimal inflammatory cell infiltration and thin fibrous membrane formation in the *Wnt3a* treatment group at 12 weeks, with no significant bone destruction observed. By 16 weeks, a relative decrease in inflammatory cells and reduced membrane thickness was noted, indicative of a positive therapeutic response (Figure 2B).

Given the critical roles of OPG and OCN in osteoblast differentiation and bone development, we assessed their expression levels following *Wnt3a* overexpression. Western blot analysis confirmed an increase in both OPG and OCN levels (Figures 3B,C; Supplementary Figure S2). Consistent with these findings, a panel of osteogenic genes, including *Ocn*, *Opg*, runt related transcription factor 2 (*Runx2*), alkaline phosphatase, liver/bone/kidney (*Alpl*), and transcription factor 4 (*Tcf4*), were upregulated post-*Wnt3a* overexpression (Figure 3D). In brief, these results suggest that AAV-mediated *Wnt3a* overexpression promotes osteoblast formation in prosthetic model mice.

### 3.4 Combination gene therapy of *TNF-α* knockdown and *Wnt3a* overexpression increased periprosthetic bone mass

**FIGURE 4:**
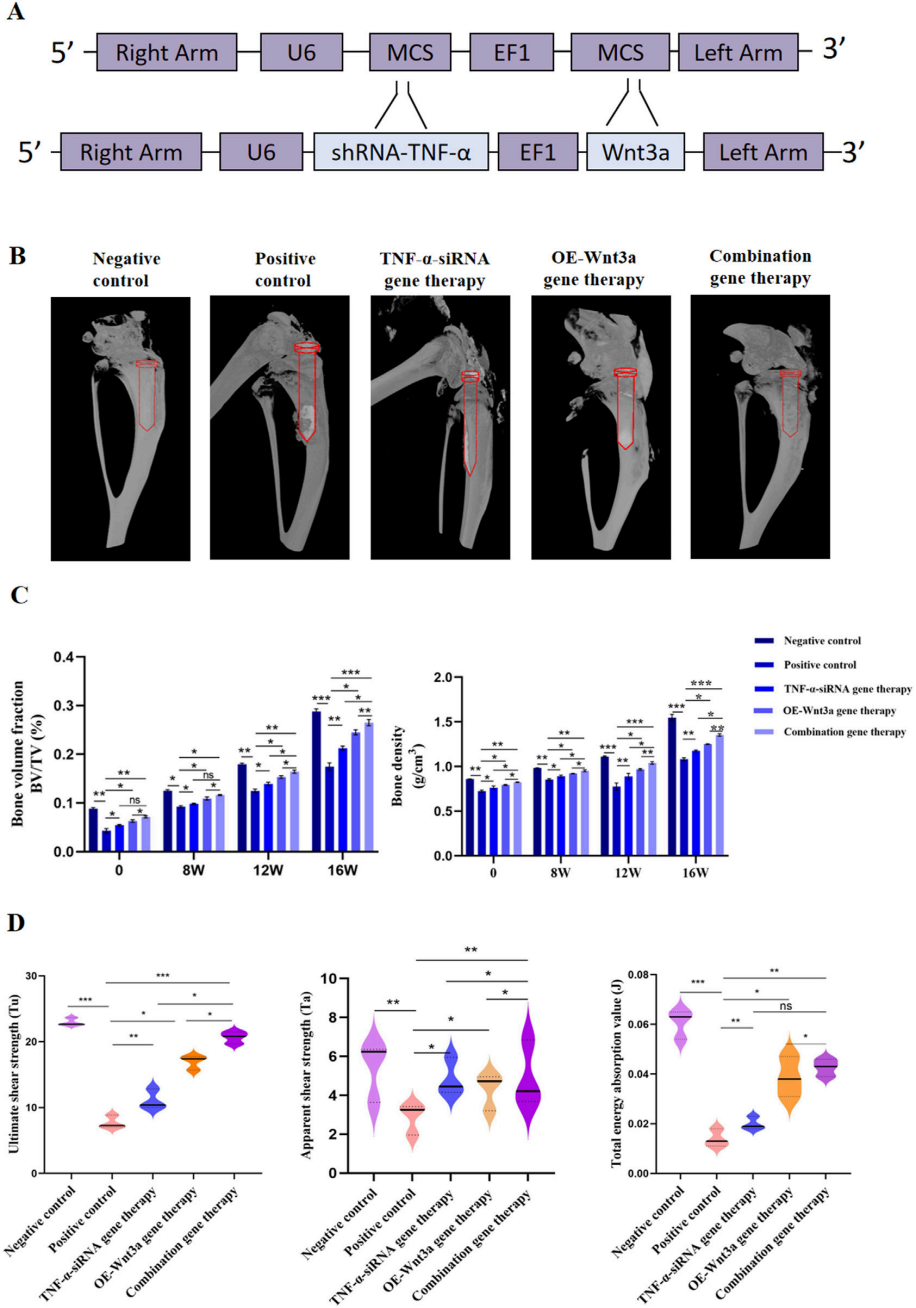
Combination gene therapy of *Wnt3a* overexpression and TNF-α knockdown increased periprosthetic bone mass **(A)** Schematic representation of adeno-associated viral vectors expressing *Wnt3a* and TNF-α-siRNA simultaneously **(B)** Three dimensional reconstruction of periprosthetic bone at week 16 **(C)** Quantitative analysis of bone volume fraction (BV/TV) and bone mineral density (BMD) under different times **(D)** Ultimate shear strength, Apparent shear strength and Total energy absorption value identified by biomechanical evaluation after 16 weeks intervention period.

The above findings demonstrated the therapeutic effects of *TNF-α*-siRNA and *Wnt3a* gene therapies individually. Building on these results, we investigated the synergistic potential of combining these two therapeutic strategies for the treatment of aseptic loosening. We constructed an AAV vector carrying both the *TNF-α* interference and *Wnt3a* overexpression sequences (Figure 4A). ELISA assays confirmed significant downregulation of *TNF-α* in the combination gene therapy group, comparable to the *TNF-α*-siRNA monotherapy group and markedly lower than the positive control group (Figure 2A). Interestingly, at 16 weeks, the combination gene therapy group showed even lower *TNF-α* expression than the *TNF-α*-siRNA monotherapy group (Figure 2A). Concurrently, the combination gene therapy group exhibited *Wnt3a* expression levels similar to the *Wnt3a* monotherapy group, both significantly higher than the positive control group, with elevated *Wnt3a* expression observed as early as 12 weeks in the combination group (Figure 3A). These results indicate the successful development of an AAV vector capable of knocking down *TNF-α* and overexpressing *Wnt3a*.

The combination gene therapy group demonstrated thinner boundary membranes, reduced inflammatory cell infiltration, and enhanced bone formation at 12 weeks compared to the single gene therapy groups. Over the course of treatment, the combination gene therapy group showed superior therapeutic effects, with significant reductions in RANKL and TRAP expression and increases in OCN and OPG protein levels compared to the control group (Figures 2C, D). Notably, differential expression levels of RANKL, OCN, and OPG genes were observed between the combination and single gene therapy groups (Figures 2C, D). Consistent with these findings, the combination gene therapy group had a lower number of TRAP-positive cells (Figures 2E, F).

Micro-CT scanning and three-dimensional reconstruction of the mouse knee prosthesis revealed pronounced osteolysis in the positive control group, while the gene therapy groups showed significantly reduced osteolysis compared to the positive control. The combination gene therapy group had significantly higher bone volume fraction and bone density than the single gene therapy group (Figures 4B, C). Biomechanical testing further supported our primary findings, with the ultimate shear strength value in the positive control group being lower than the negative control group, indicating prosthesis loosening post-implantation (Figure 4D). Gene therapy partially restored this value, with the combination gene therapy group showing higher ultimate shear strength, apparent shear stiffness, and total energy absorption values compared to the single gene therapy group, further highlighting the efficacy of the combination gene therapy.

### 3.5 Combination gene therapy of *TNF-α* knockdown and *Wnt3a* overexpression could effectively alleviate bone resorption

**FIGURE 5:**
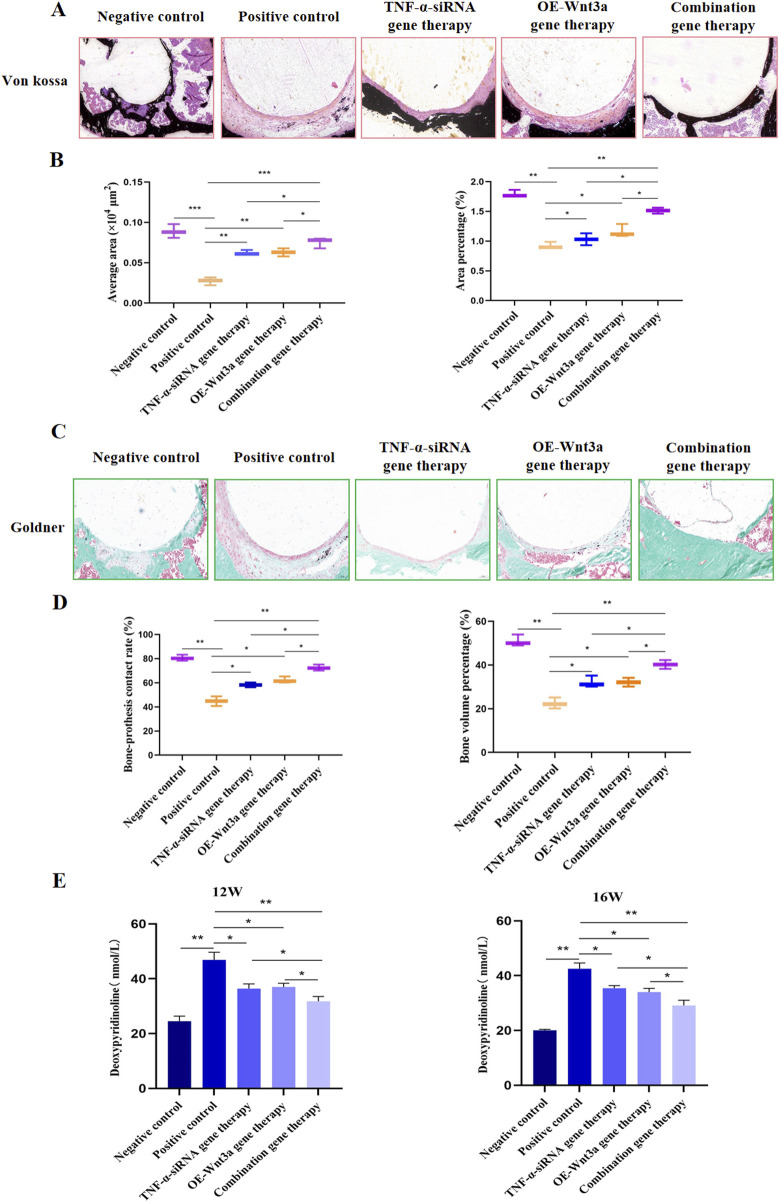
Combination gene therapy of *Wnt3a* overexpression and TNF-α knockdown could effectively alleviate bone resorption **(A)** Von Kossa staining of proximal tibia at 16 weeks postoperatively **(B)** The average area and Area fraction of calcium salt deposition islands in the cortical bone at 16 weeks postoperatively **(C)** Goldner staining of proximal tibia at 16 weeks postoperatively **(D)** Quantification of bone prosthesis contact rate and bone volume percentage at week 16 **(E)** The levels of deoxypyridinoline in the urine after 12-week and 16-week intervention period.

The therapeutic efficacy of combination gene therapy targeting *TNF-α* knockdown and *Wnt3a* overexpression was further evaluated at the 16-week mark, with a focus on its ability to alleviate bone resorption. Von Kossa staining was utilized to visualize calcium deposits, revealing more pronounced dark black staining in the negative control group, indicating robust mineralization. In contrast, the positive control group showed less black-brown staining, suggesting reduced mineral deposition and increased bone resorption due to the influence of titanium particles on bone metabolism (Figure 5A). Post-gene therapy, a significant enhancement in mineralization was observed, with quantitative analysis showing a substantial difference in the mean area of calcium salt deposition islands between the single gene therapy group and the positive control group (Figure 5B). Notably, the combination gene therapy group exhibited a significantly greater mean area of calcium salt deposition islands compared to the single gene therapy group, and a similar trend was observed in the quantification of the area fraction of calcium salt sedimentary islands.

To gain a comprehensive understanding of the gene therapy’s impact, Goldner trichrome staining was performed (Figure 5C). The negative control group displayed a greater proportion of green-stained areas, indicating less mineralization, while the positive control group showed a higher proportion of orange-red stained areas, suggesting severe non-mineralization. Image analysis using IPP software was conducted to quantify the bone-implant contact rate and bone volume fraction based on the staining results. The average bone-prosthesis contact rate in the positive control group was below 50%, whereas the gene therapy group showed a contact rate exceeding 80% (Figure 5D). The combination gene therapy group demonstrated an even higher bone-prosthesis contact rate compared to the single gene therapy group. Additionally, the bone volume percentage surrounding the prosthesis in the gene therapy group significantly increased compared to the control group, with the combination gene therapy group showing an even higher value.

Furthermore, we assessed urinary DPD levels, a well-recognized biomarker of collagen degradation released during osteoclast-mediated bone resorption, to complement our histological staining. The urine DPD concentrations in the positive control group were significantly elevated compared to the negative control group. Following gene therapy, a reduction in DPD levels was observed, with the combination gene therapy group showing a particularly pronounced decrease (Figure 5E). These findings, along with the DPD data, collectively demonstrate that the combination gene therapy involving *Wnt3a* overexpression and *TNF-α* knockdown can effectively mitigate bone resorption, offering a promising approach for the treatment of periprosthetic osteolysis.

### 3.6 Safety assessment of gene therapy

**FIGURE 6:**
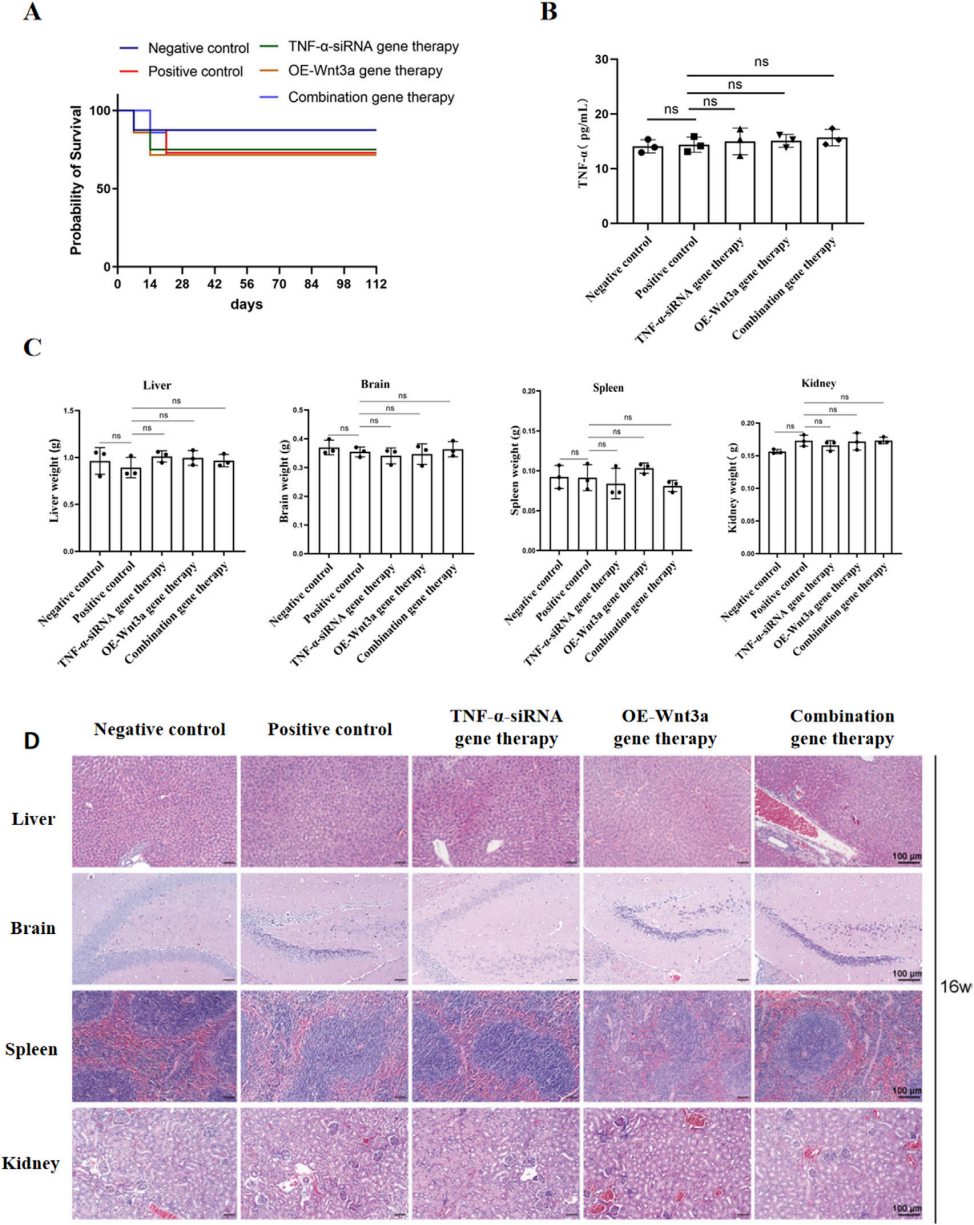
Safety assessment of gene therapy **(A)** Survival curve of mice after gene therapy **(B)** Detection of TNF-α expression in peripheral blood by ELISA **(C)** Weight of major organs including heart, kidney, spleen, liver **(D)** Representative H&E-stained slices of major organs including heart, kidney, spleen, liver.

To ensure a thorough assessment of the safety profile of our gene therapy approach, we conducted a series of evaluations monitoring mouse survival and potential systemic effects. Initially, we tracked the survival status of the mice at various time points post-treatment. Figure 6A illustrates that while a few early deaths occurred post-modeling, the implementation of gene therapy did not lead to any additional increase in mortality, suggesting that our gene therapy strategy did not adversely impact the overall survival rate of the mice. Furthermore, to determine if gene therapy might induce a systemic inflammatory response, we quantified the levels of the inflammatory cytokine *TNF-α* in peripheral blood (Figure 6B). The data revealed no significant differences in *TNF-α* levels across the experimental groups, with all maintaining low *TNF-α* levels, thereby confirming that gene therapy did not trigger significant systemic inflammatory responses.

After a 16-week treatment period, mice were euthanized, and organs including the heart, brain, spleen, and liver were harvested and weighed. The organ weights showed no significant differences between the treatment and control groups, with all organs appearing normal in size (Figure 6C). Subsequent histological examination using H&E staining was performed on these major organs to assess tissue morphology. The histological analysis demonstrated preserved normal tissue architecture across all examined organs, with prominent nucleoli and no observable pathological abnormalities (Figure 6D). Collectively, these findings suggest that the intra-articular administration of the adenoviral vector into the knee joint cavity does not induce systemic toxicity in other organs, supporting the safety of our gene therapy approach.

## 4 Discussion

Periprosthetic osteolysis, triggered by wear particles from joint replacements, remains a critical clinical challenge that often leads to implant failure and the need for revision surgery (Gallo et al., 2014). Despite advancements in implant materials and designs, there is an unmet need for effective strategies to prevent this condition and prolong implant longevity. Our study presents a novel approach by demonstrating that the combined intervention of *TNF-α* knockdown and *Wnt3a* overexpression significantly mitigates osteoclast-related gene expression, enhances osteoblast-associated genes, and improves bone density and biomechanical properties around the prosthesis, offering a promising therapeutic strategy for periprosthetic osteolysis.

The pathophysiology of periprosthetic osteolysis is characterized by an imbalance in bone homeostasis around the prosthesis (Wang et al., 2023; Zhang et al., 2020). In our study, the suppression of *TNF-α* expression via AAV-mediated siRNA delivery significantly reduced osteoclastogenesis, as evidenced by decreased levels of osteoclast-related markers such as RANKL and TRAP (Figures 2C, D). This finding aligns with previous studies demonstrating the critical role of *TNF-α* in promoting osteoclast differentiation and bone resorption (Fan et al., 2023; Ma et al., 2023). The reduction in TRAP-positive cells following *TNF-α* knockdown further supports the critical role of TRAP in osteoclast-mediated bone resorption. TRAP, encoded by the *Acp5* gene, is a well-established marker of osteoclast activity and has been implicated in various bone disorders, including spondyloenchondrodysplasia (Ramesh et al., 2020). In addition, *TNF-α* is known to upregulate RANKL expression, which is essential for osteoclast formation and activation (Komine et al., 2001). By targeting *TNF-α*, we effectively disrupted this pathway, leading to a reduction in osteoclast activity and bone resorption.

Simultaneously, *Wnt3a* overexpression increased the expression levels of osteoblast-associated genes, including OPG and OCN (Figures 3B, C), aligning with existing literature that emphasizes the bone-protective effects of *Wnt3a* (Hamamura et al., 2014; Xiong et al., 2024). The *Wnt/β-catenin* signaling pathway, activated by *Wnt3a*, plays a pivotal role in promoting osteoblast differentiation and bone formation (Li et al., 2022). By overexpressing *Wnt3a*, we enhanced osteoblastic activity, leading to increased bone formation and mineralization, as demonstrated by the improved bone density and biomechanical properties observed in our study (Figures 4B–D).

The synergistic effect of our combination gene therapy enhanced therapeutic efficacy beyond that of monotherapy, effectively mitigating osteolysis by reducing inflammatory responses and simultaneously promoting bone formation. Biomechanical analysis and histological staining confirmed increased bone density and improved prosthesis-bone integration in the combination gene therapy group (Figures 4B–D). This synergistic effect can be attributed to dual targeting of the inflammatory and anabolic pathways involved in periprosthetic osteolysis, aligning with the current understanding of the disease’s pathophysiology, where both inflammatory and anabolic imbalances contribute to disease progression (Crotti et al., 2015; Panez-Toro et al., 2023; Yu et al., 2020).

The enhanced efficacy of combined *TNF-α* knockdown and *Wnt3a* overexpression is rationalized by the intricate interplay between inflammation and bone metabolism (Stavre et al., 2023). *TNF-α*, a central mediator of inflammation, is known to inhibit bone formation by suppressing the Wnt/β-catenin signaling pathway, crucial for osteoblast differentiation and function (Hu et al., 2021; Yarilina et al., 2011). By knocking down *TNF-α*, we not only reduced the inflammatory response but also alleviated its suppressive action on osteogenic processes, creating a more conducive environment for bone anabolism. Concurrently, *Wnt3a* overexpression stimulated the Wnt/β-catenin pathway, leading to heightened osteoblast activity and bone formation, particularly effective in the anti-inflammatory milieu established by *TNF-α* knockdown (Hu et al., 2021; Jiao et al., 2023). This dual targeting strategy addresses the key pathogenic mechanisms of periprosthetic osteolysis more comprehensively than either intervention alone.

Furthermore, our research underscores the potential of gene therapy as a precise and potent treatment modality. The localized delivery of therapeutic genes directly to the site of implantation minimizes systemic side effects and maximizes therapeutic impact, crucial given the focal nature of periprosthetic bone loss and the desire to avoid broad immunosuppression or off-target effects. Our study utilized adenovirus-mediated gene therapy, which has shown promise in preclinical studies, but its efficacy in clinical trials needs further verification. While we focused on *TNF-α* and *Wnt3a*, other cytokines and signaling pathways may also play significant roles in periprosthetic osteolysis (Liao et al., 2019; Liu et al., 2022; Wang et al., 2018; Yu et al., 2024). Future studies should consider the broader inflammatory and anabolic milieu to develop a more comprehensive therapeutic strategy.

In conclusion, the combination gene therapy targeting *TNF-α* and *Wnt3a* simultaneously holds significant promise for the treatment of wear particle-induced periprosthetic osteolysis. This synergistic intervention aligns with the complex pathophysiology of periprosthetic osteolysis and offers a novel avenue for therapeutic development in joint replacement surgeries.

## Data Availability

The original contributions presented in the study are included in the article/Supplementary material, further inquiries can be directed to the corresponding authors.
