# Supplementary figures and images for: Combining TNF-α silencing with Wnt3a overexpression: a promising gene therapy for particle-induced periprosthetic osteolysis
Source: Front Cell Dev Biol. 2025 Mar 6;13:1511577. doi: 10.3389/fcell.2025.1511577 (PMC11922860; doi:10.3389/fcell.2025.1511577)

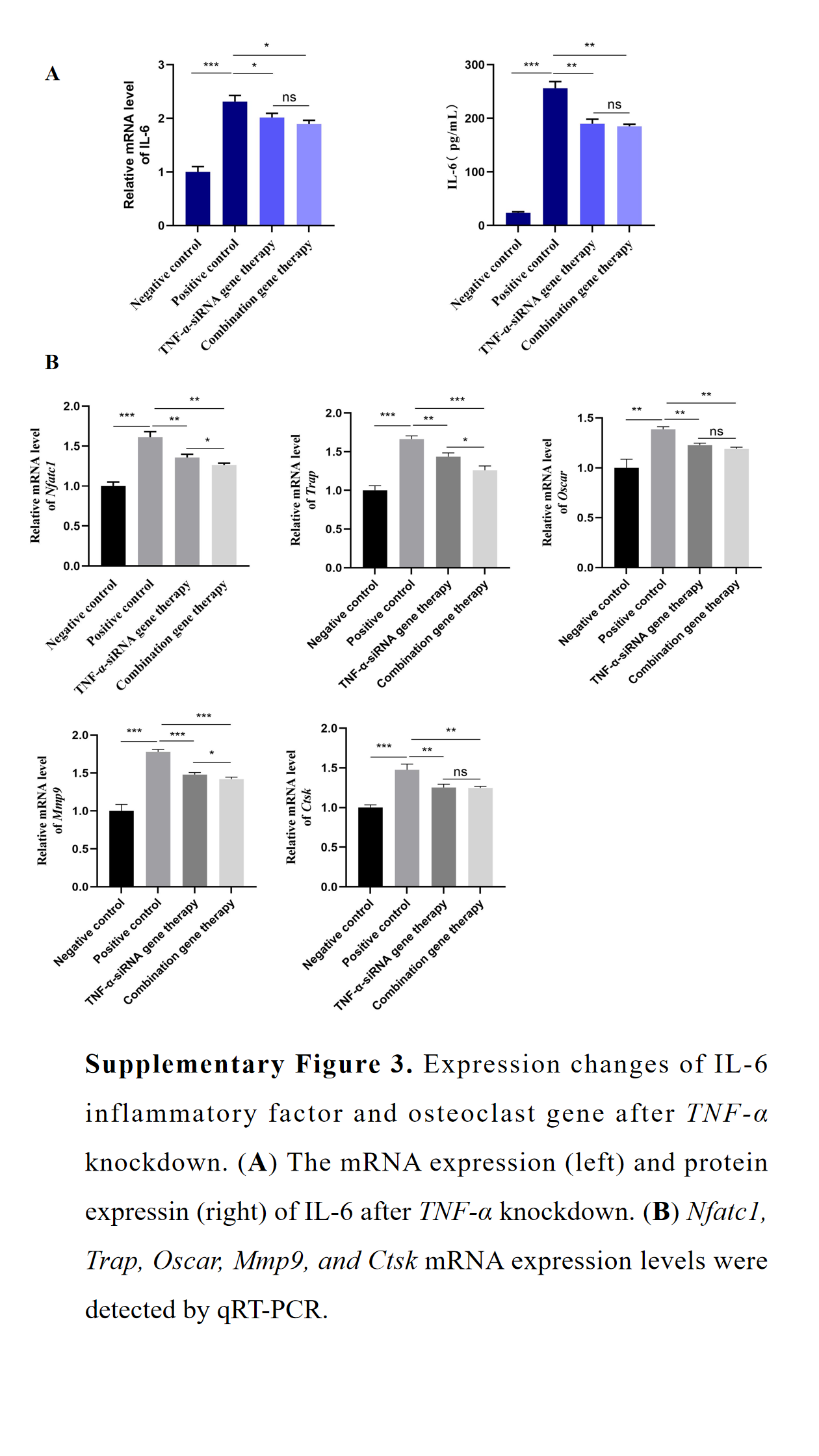

Supplement: Supplementary file 1 [file Image3.tif]

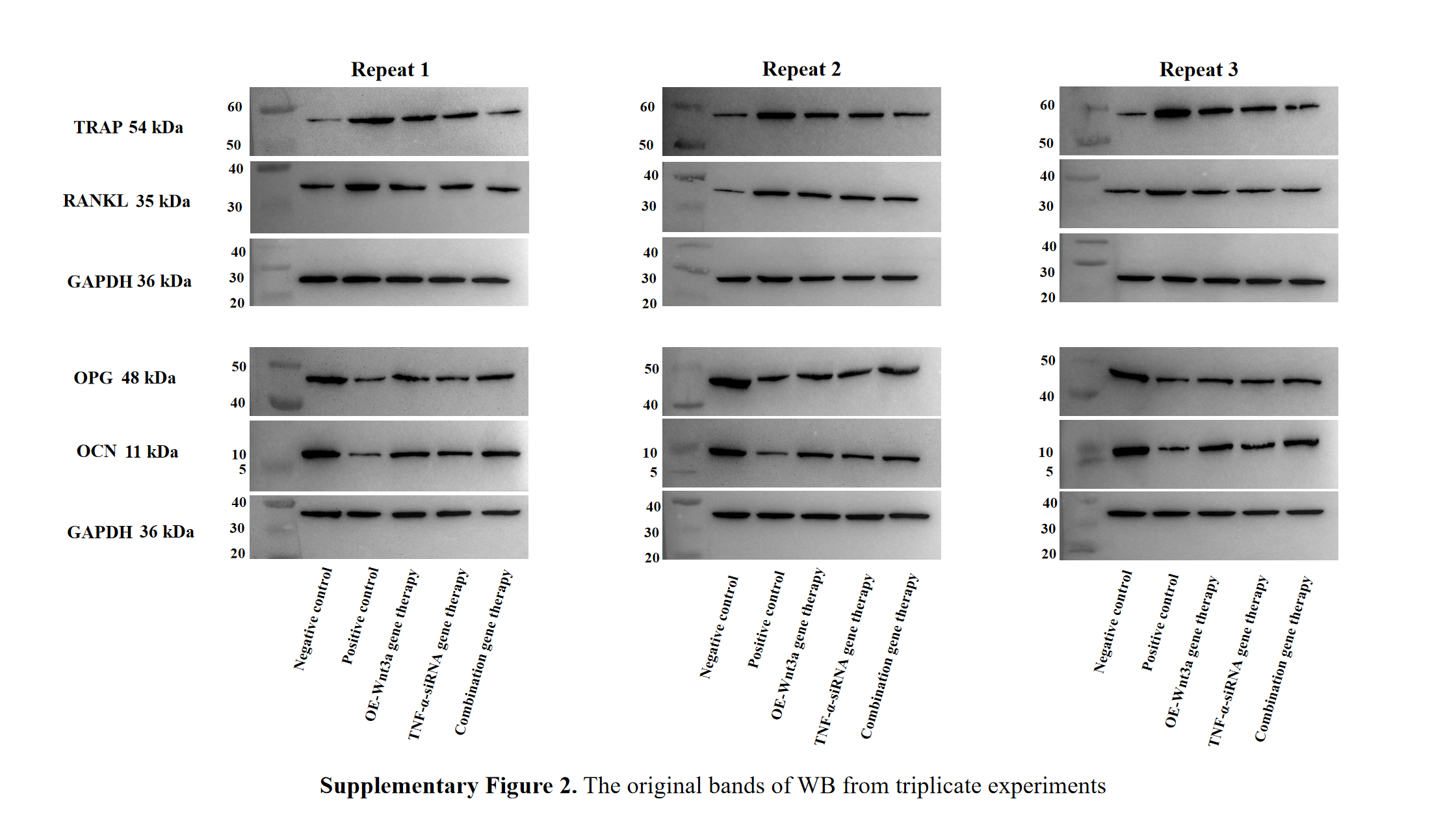

Supplement: Supplementary file 2 [file Image2.tif]

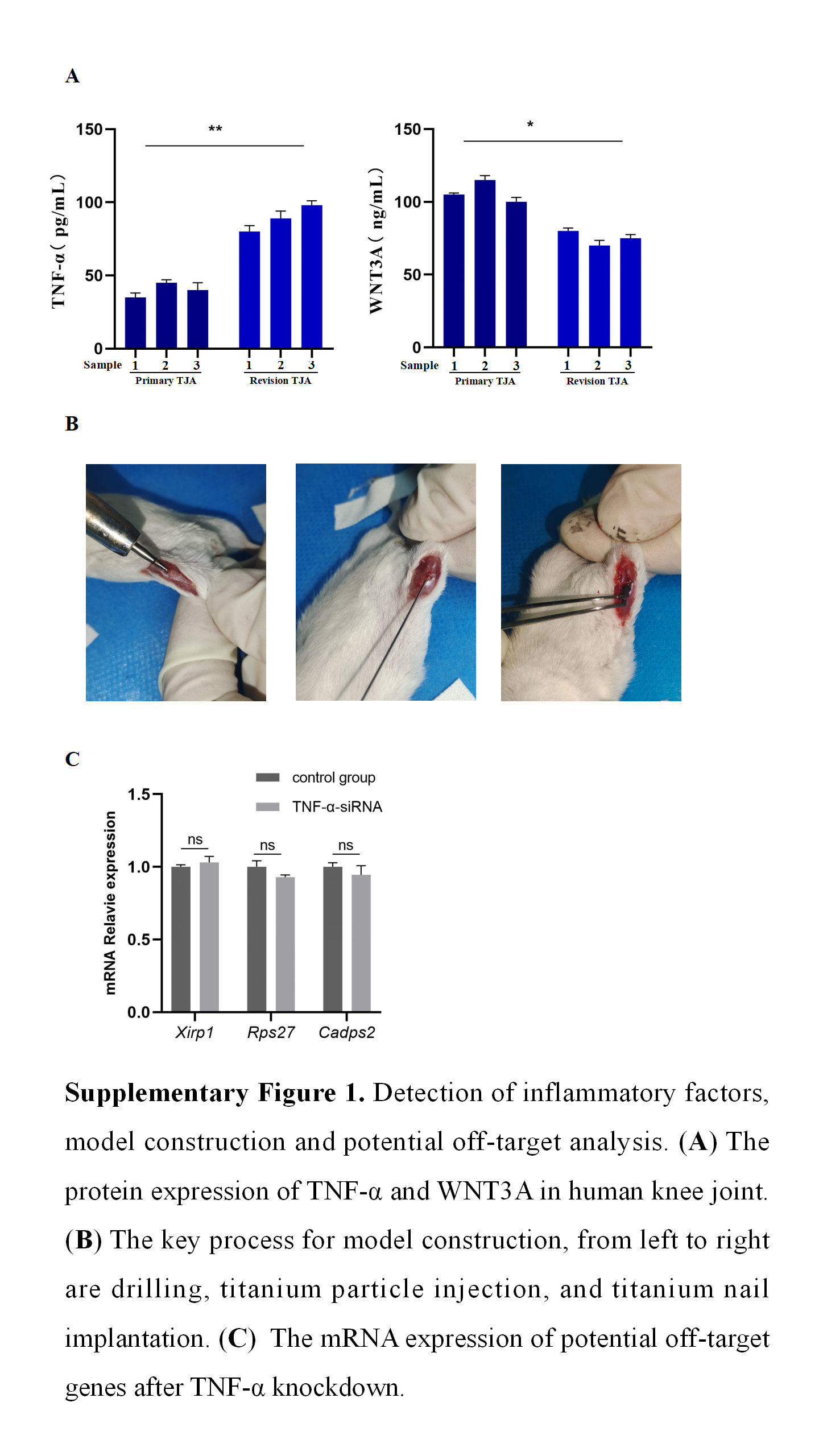

Supplement: Supplementary file 3 [file Image1.tif]
